# ‘One Stop’ Therapy has a Satisfying Performance on AF Patients with Interatrial Communication: Evidence from Pooled Clinical Experience

**DOI:** 10.31083/RCM26662

**Published:** 2025-04-16

**Authors:** Zhi-Yuan Zhang, Feng Li, Chi Geng, Yu-Qi Chen, Si-Liang Peng, Yao-Ting Zhang, You Zhang, Xiao-Song Gu, Hui Li

**Affiliations:** ^1^Department of Cardiology, The Second Affiliated Hospital of Soochow University, 215004 Suzhou, Jiangsu, China

**Keywords:** atrial fibrillation, atrial septal defect closure, ASD closure, patent foramen ovale closure, PFO closure, left atrial appendage closure, LAAC

## Abstract

**Background:**

Left atrial appendage closure (LAAC) has been reported to be a viable alternative to prevent thromboembolic events for atrial fibrillation (AF) patients. Interatrial communication closure, such as atrial septal defect (ASD) and patent foramen ovale (PFO) closure could significantly decrease the occurrence of stroke. For AF patients with interatrial communication, the success rate as well as the long-term outcomes of ‘One stop’ closure remain elusive.

**Methods:**

Studies were systematically screened using online databases (including PubMed, Cochrane Library, Web of Science, Embase, China National Knowledge Infrastructure (CNKI) database, and WanFang database) from their establishment to 1st August 2024. We utilized a fixed-effect model to synthesize the success rate and the long-term outcomes. Subgroup analysis was performed to identify the potential confounders.

**Results:**

A total of 7 studies comprising 156 patients were included. ASD/PFO closure combined with LAAC showed a high degree of feasibility, with a success rate of 1.00 (95% CI: 0.99, 1.00; *p* < 0.001). Meanwhile, ‘One stop’ ASD/PFO closure combined with LAAC exhibited a high long-term safety and a low occurrence of complications. Moreover, subgroup analysis revealed that the bleeding event occurrence was relatively higher in the male proportion ≥50% subgroup and HAS-BLED score ≥3 subgroup, respectively.

**Conclusions:**

ASD/PFO closure combined with LAAC has a satisfying performance on AF patients with interatrial communication.

**The PROSPERO Registration:**

CRD42023462221, https://www.crd.york.ac.uk/prospero/display_record.php?ID=CRD42023462221.

## 1. Introduction

Interatrial communication is one of the most common congenital heart malformations, including atrial septal defect (ASD) and patent foramen ovale (PFO) [1]. It is characterized by a deficiency in the septum that separates the two atria, allowing for a direct connection between the two atria and facilitating blood flow from the left to the right atrium [2]. ASD is typically categorized into a secundum, primum, sinus venosus, or coronary sinus defect. Among these, the ostium secundum atrial septal defect is the most prevalent, constituting roughly 7% of all congenital heart malformations [3]. In most cases, children and teenagers with ASD are symptom-free, but over time, complications such as arrhythmia, thromboembolism, pulmonary arterial hypertension and even right heart failure may occur. In addition, PFO is even more widespread, with a prevalence ranging from 25% to 27% among adults in the general population. Previous studies indicated PFO was associated with strokes, migraines, and platypnea-orthodeoxia syndrome, even leading to cryptogenic embolic strokes in young patients [4]. Numerous randomized controlled trials have indicated that, compared with oral anticoagulant and antiplatelet therapy, effective ASD/PFO closure can significantly reduce the occurrence of complications such as stroke [5, 6].

Atrial fibrillation (AF) is the most prevalent arrhythmia worldwide, and its increasing prevalence, driven by increased life expectancy, represents a significant public health challenge [7]. AF markedly deteriorates quality of life and is associated with severe complications, including stroke, heart failure, cognitive impairment, and cardiac arrest [8, 9]. Currently, oral anticoagulants (OACs) have been recommended as the first-line treatment for preventing thromboembolism in AF patients. Whereas, emerging studies have demonstrated that left atrial appendage closure (LAAC) can serve as an alternative to anticoagulant therapy for patients who are intolerant of oral anticoagulants, significantly reducing the incidence of cardiogenic stroke events in AF patients [10, 11].

Interestingly, ASD/PFO closure and LAAC both belong to the transarterial septal operation, allowing for the combination of the two interventions (‘One stop’ therapy) to be an available and practical approach. Whereas, previous studies may fail to provide reliable and comprehensive conclusions about the feasibility, efficacy and safety of ‘One stop’ therapy due to some limitations, such as lack of long-term follow-up and small sample size. Therefore, we performed this meta-analysis with the aim of further evaluating the performance of ‘One stop’ therapy on patients with interatrial communication and AF, and to screen for potential determinants for ‘One stop’ therapy.

## 2. Methods

### 2.1 Study Design

This study protocol, registered in the PROSPERO database (CRD42023462221), was developed in accordance with the PRISMA guidelines.

### 2.2 Search Strategy

Two independent reviewers (ZYZ and FL) performed an extensive search on online databases, such as PubMed, Cochrane Library, Web of Science, Embase, WanFang and China National Knowledge Infrastructure (CNKI) database, from their establishment to 1st August 2024. Search keywords included “atrial fibrillation”, “AF”, “non-valvular atrial fibrillation”, “NVAF”, “left atrial appendage closure”, “left atrial appendage occlusion”, “LAAC”, “LAAO”, “PFO closure”, “patent foramen ovale closure”, “ASD closure” and “atrial septal defect closure”. Also, we conducted a manual search of the reference lists in the reviewed literature and retrieved eligible literature to identify potential publications that may have been overlooked. Specific search strategies are described in **Supplementary Material 1**.

### 2.3 Search Design

The titles, abstracts, and full texts were comprehensively searched and evaluated by two independent raters, ZYZ and FL, to identify eligible studies. Studies were eligible if they met the following inclusion criteria: (1) Randomized controlled trials and cohort, observational studies, and case-control studies; (2) Studies reporting the efficacy and safety of combining ASD/PFO closure with LAAC in the AF patients with interatrial communications. (3) English or Chinese studies published in peer-reviewed journals with full text available. (4) In cases of multiple articles on the same trial or cohort, only the study with the largest data volume was included. Studies without original data, animal studies, reviews, case reports, letters, and editorials were excluded. A third reviewer (HL) was involved in resolving any disputes related to eligibility.

### 2.4 Data Extraction and Quality Assessment

For each eligible study, data were extracted independently by two researchers (ZYZ and FL), and any potential disagreements were resolved through discussion with a third investigator (HL). Initially, we extracted the study characteristics, including publication year, study design, primary author, patient count, and follow-up duration. Additionally, we recorded the demographic and clinical characteristics of patients, the criteria for implementing the one-stop procedure, the devices utilized during the intervention, the postoperative antithrombotic regimens and the long-term efficacy and safety outcomes. The potential for bias in each eligible study was evaluated separately by two researchers (SLP and YTZ) utilizing the Newcastle Ottawa Quality Assessment Scale [12].

### 2.5 Statistical Analysis

Statistical analyses were conducted using Stata, version 16.0 (Stata Corp LP, College Station, TX, USA). Continuous variables were displayed as means ± standard deviations and categorical variables were showed as frequencies or percentages. Pooled results were described with event rates (eg. the ratio of the number of events to the number of patients) and their corresponding 95% confidence intervals. The I^2^ index was utilized to quantify the extent of heterogeneity in quantitative studies [13], with the ranges of 0% (no heterogeneity), <25% (low heterogeneity), 25%–49% (moderate heterogeneity), and >50% (high heterogeneity). In cases where the I^2^ value exceeded 50%, significant heterogeneity among studies was considered present and analysis was performed using a random effects model. If this threshold was not reached, a fixed effects model was employed instead. Sensitivity analyses would be conducted in instances of significant heterogeneity to assess the impact of individual studies on overall risk estimates by excluding each study one by one. Furthermore, potential publication bias would be evaluated with Egger’s tests. *p* < 0.05 represented a statistically significant difference.

The study also conducted subgroup analyses to investigate the sources of heterogeneity. In line with previously reported factors as well as the characteristics of eligible studies, seven subgroup factors were screened, including the sample size (≥20 vs. <20), the interatrial communications types (ASD and PFO vs. ASD vs. PFO), age (≥60 years vs. <60 years), male proportion (≥50% vs. <50), CHA_2_DS_2_-VASc score (≥2 vs. <2), HAS-BLED score (≥3 vs. <3), and follow-up (>12 months vs ≤12 months).

## 3. Results

### 3.1 Study Selection and Quality Assessment

**Fig. 1.**
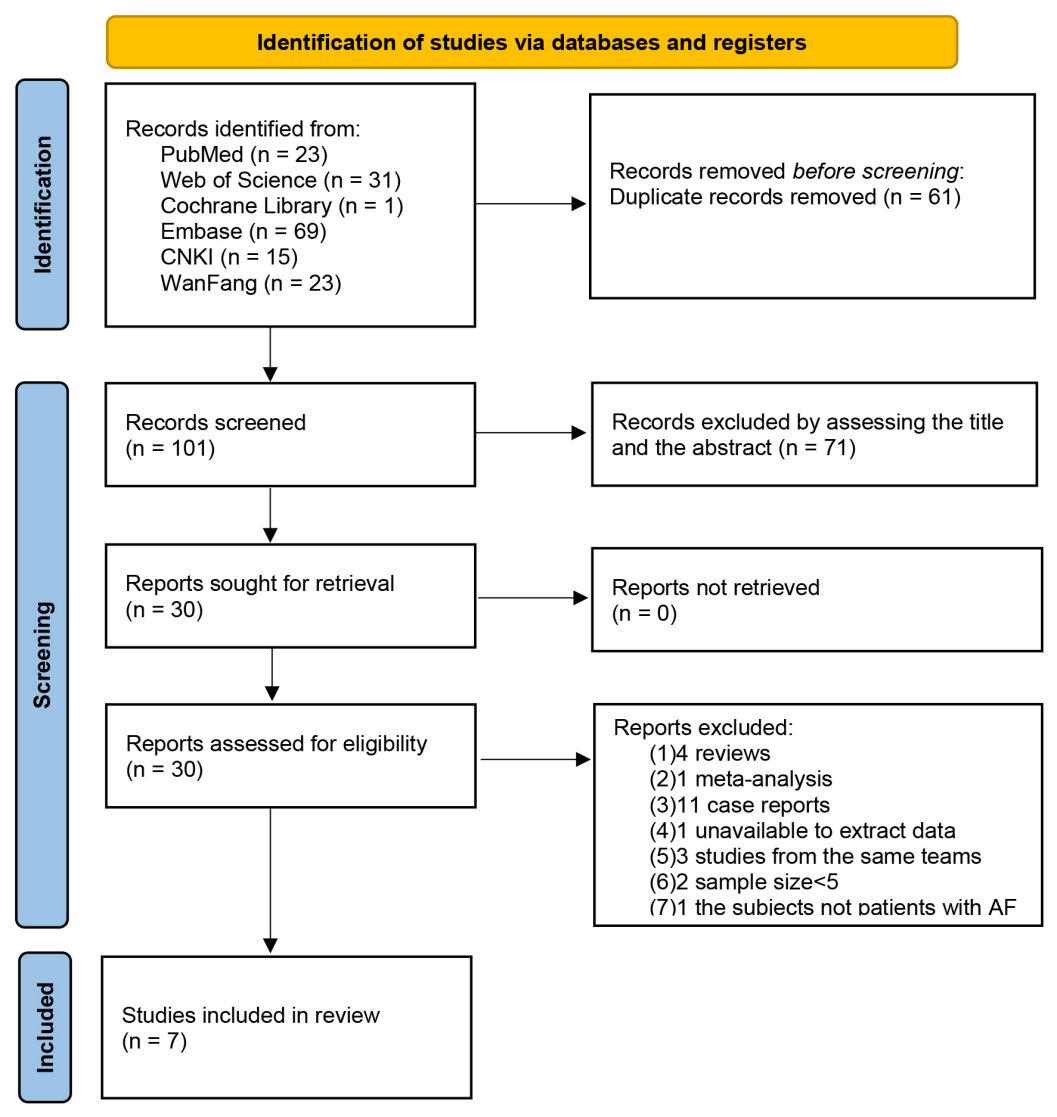
**The flowchart of the study selection**. AF, atrial fibrillation; CNKI, China National Knowledge Infrastructure.

After preliminary screening, a total of 162 articles met the inclusion criteria for the study. Following removal of duplicate entries and screening all titles and abstracts, 30 articles were retained for further evaluation. Next, we removed 23 additional articles by reading the full text of the remaining 30 articles. Among them, 4 reviews, 1 meta-analysis, 11 case reports and 4 research study was deleted if data extraction wasn’t possible, or if the article was from the same team. The articles with sample sizes of less than 5 and subjects without AF were also excluded. Finally, a total of 7 articles were eligible [14, 15, 16, 17, 18, 19, 20]. The flow chart of literature screening is displayed in Fig. 1. A total of 156 patients were included in the meta-analysis. The baseline data of the patients are shown in Table 1 (Ref. [14, 15, 16, 17, 18, 19, 20]). Simultaneously, we conducted a comprehensive summary of the eligible studies, elucidated the criteria for implementing the one-stop procedure, the devices utilized during the intervention, and outlined the postoperative antithrombotic regimens (**Supplementary Material 2**).

**Table 1.** **Baseline characteristics of the patients enrolled in the eligible studies**.

First author (year)	Study design	Sample size	Male (%)	Age (years)	Persistent AF (%)	CHA_2_DS_2_-VASc score	HAS-BLED score	Follow-up (months)
Yu (2019) [14]	Retrospective study	30	86.7	75.4 ± 7.3	80.0	3.8 ± 1.6	3.7 ± 1.1	23.8 ± 16
Zhang (2020) [15]	Retrospective study	49	44.9	65.6 ± 9.6	91.8	3.5 ± 0.8	2.6 ± 0.6	29.0 ± 12.1
Cui (2016) [16]	Retrospective study	7	85.7	48.6 ± 10.0	100	1.3 ± 0.5	3.3 ± 0.5	1.5 (1–3)
Wang (2018) [17]	Retrospective study	18	44.4	56.3 ± 6.9	88.9	2.4 ± 0.8	3.1 ± 0.5	12.3 (6–19.2)
Jiang (2020) [18]	Retrospective study	13	38.5	64.8 ± 14.1	100	3.2 ± 0.9	2.2 ± 1.1	1.5 (1–3)
Zhao (2022) [19]	Retrospective study	7	28.6	68.1 ± 10.0	71.43	5.1 ± 1.4	3.0 ± 1.0	12 (1–24)
Fan (2023) [20]	Retrospective study	32	34.3	68.2 ± 9.3	90.6	2.7 ± 1.6	2.1 ± 1.1	6 (1–12)

AF, atrial fibrillation.

In addition, we conducted a quality assessment of the eligible articles, and found that all included studies presented as moderate-to-high quality (Table 2, Ref. [14, 15, 16, 17, 18, 19, 20]). All subjects in the study were representative of the population. For instance, patients encompassed were diagnosed with nonvalvular AF and ASD/PFO, and required treatment for PFO/ASD closure. Moreover, the initial outcome of interest was non-existent at the beginning of the study. In addition, except for one study with a follow-up rate below 95%, all other studies had complete follow-up [17]. However, only three studies had a follow-up of more than 12 months [14, 15, 17].

**Table 2.** **Quality evaluation of enrolled studies based on the Newcastle-Ottawa Quality Assessment Scale (NOS)**.

First author (year)	Representativeness^𝐚^	Selection of non-exposed^𝐛^	Ascertainment of exposure^𝐜^	Incident disease^𝐝^	Comparability^𝐞^	Assessment of outcome^𝐟^	Length of follow-up^𝐠^	Adequacy of follow-up^𝐡^
Yu (2019) [14]	A	A	A	A	A	B	A	A
Zhang (2020) [15]	A	A	A	A	A	B	A	A
Cui (2016) [16]	A	A	A	A	A	B	B	A
Wang (2018) [17]	A	A	A	A	A	B	A	C
Jiang (2020) [18]	A	A	A	A	A	B	B	A
Zhao (2022) [19]	A	A	A	A	A	B	B	A
Fan (2023) [20]	A	A	A	A	A	B	B	A

^𝐚^ A: truly representative; B: somewhat representative; C: selected group of users; D: no description of the derivation of the cohort. ^𝐛^ A: sourced from the same community as the exposed cohort; B: sourced from a different source; C: no details on the derivation of the non-exposed cohort. ^𝐜^ A: securely recorded data; B: structured interview conducted; C: written self-report provided; D: no description given. ^𝐝^ Demonstration that outcome of interest was absent at study commencement. A: yes; B: no. ^𝐞^ A: study controls for demographics/clinical characteristics; B: study controls for any additional factor (e.g., age, HAS-BLED and CHA_2_DS_2_-VASc score); C: not performed. ^𝐟^ A: independent blind assessment; B: record linkage; C: self-report; D: no description. ^𝐠^ Was follow-up duration sufficient to capture outcomes? A: yes (i.e., the mean follow-up approximately 12 months or longer); B: no. ^𝐡^ A: complete follow-up; B: subjects lost to follow-up was unlikely to introduce bias; C: follow-up rate below 95% or lower observed; D: no statement.

### 3.2 Success Rate of ‘One Stop’ ASD/PFO Closure Combined with LAAC

**Fig. 2.**
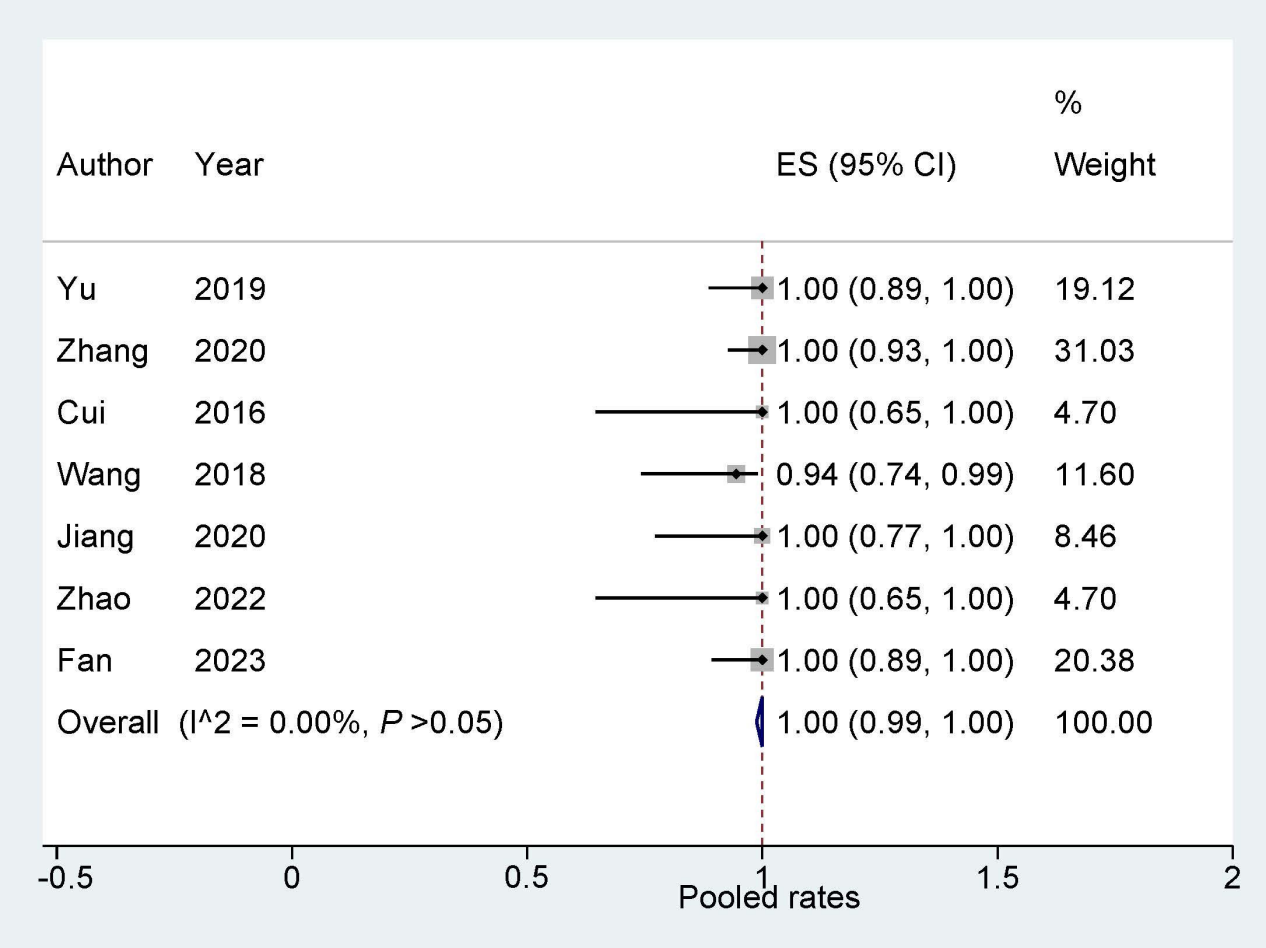
**Success rate of ‘One stop’ ASD/PFO closure combined with LAAC**. ASD, atrial septal defect; PFO, patent foramen ovale; LAAC, left atrial appendage closure; ES, effect size.

All 7 studies [14, 15, 16, 17, 18, 19, 20] have reported the success rate of ASD/PFO closure combined with LAAC. These data were subjected to analysis using a fixed effects model. The findings reveal that the integrated interventional approach of ASD/PFO closure combined with LAAC displays a high degree of feasibility, demonstrating a success rate of 1.0 (95% confidence interval: 0.99, 1.00; *p* < 0.001; I^2^ = 0.00%, Fig. 2). In addition, we also carried out a sensitivity analysis, and the results indicated that there was no significant change in the overall combined proportion, with the range being from 1.00 (95% CI: 0.96, 1.03) to 1.01 (95% CI: 0.97, 1.03). This indicates that no individual study dominated the combined proportion and heterogeneity. Additionally, Egger’s test was conducted and showed no publication bias (*p* = 0.692), indicating that the results were robust.

### 3.3 Long-Term Adverse Events of ‘One Stop’ Closure ASD/PFO Closure Combined with LAAC

**Fig. 3.**
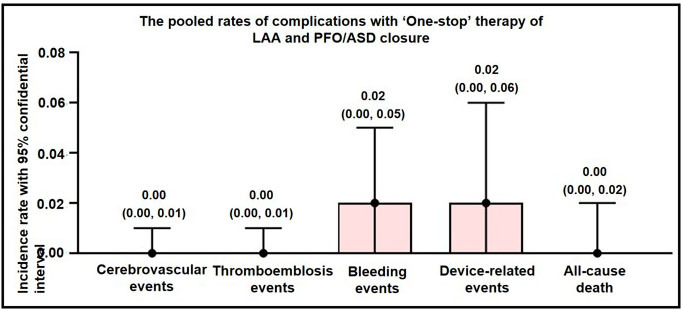
**Long-term adverse events of ‘One stop’ ASD/PFO closure combined with LAAC**. ASD, atrial septal defect; PFO, patent foramen ovale; LAAC, left atrial appendage closure; LAA, left atrial appendage.

All eligible studies had scheduled a follow-up visit with patients after interventional operation and documented safety outcomes. All long-time adverse events were analyzed using a fixed effect model. The combined rates of complications associated with the ‘One stop’ therapy of ASD/PFO closure and LAAC indicated the following: cerebrovascular events at 0.00 (95% CI: 0.00, 0.01), thromboembolic events at 0.00 (95% CI: 0.00, 0.01), bleeding events at 0.02 (95% CI: 0.00, 0.05), device-related events at 0.02 (95% CI: 0.00, 0.06), and all-cause death at 0.00 (95% CI: 0.00, 0.02) (Fig. 3).

#### 3.3.1 Bleeding Events 

**Fig. 4.**
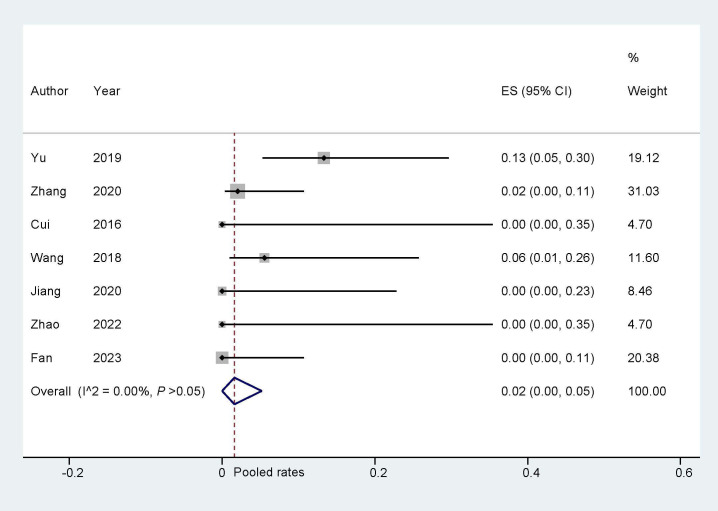
**Bleeding events during follow up**. ES, effect size.

During the follow-up of the eligible studies in this research, complications such as major bleeding, gastrointestinal bleeding, and other bleeding were observed, and were classified as bleeding events. The analysis of bleeding events indicated a pooled rate of 0.02 (95% CI: 0.00, 0.05; I^2^ = 0.00%, Fig. 4). Sensitivity analysis revealed that there was no significant change in the overall combination proportions, ranging from 0.03 (95% CI: 0.01, 0.11) to 0.11 (95% CI: 0.51, 0.24). Additionally, Egger’s test indicated no publication bias (*p* = 0.690).

Subgroup analysis was performed with seven subgroup factors for the bleeding events, and the results are displayed in Table 3. In the male proportion ≥50% subgroup, the risk of bleeding from ‘One stop’ ASD/PFO closure with LAAC was significantly higher than that of the male proportion <50% subgroup (interaction *p* = 0.045). In addition, the bleeding risk of ‘One stop’ ASD/PFO closure with LAAC was also significantly increased in the HAS-BLED score >3 subgroup (interaction *p* = 0.033).

**Table 3.** **The subgroup analysis for bleeding events**.

Factors		Study number	Pooled incidence	95% CI	I^2^ (%)	*p* value
Sample size						0.995
	≥20	3	0.03	0.00, 0.08	-	
	<20	4	0.01	0.00, 0.08	0.00%	
Interatrial communications types						0.221
	ASD and PFO	4	0.04	0.00, 0.09	0.00%	
	ASD	2	0.00	0.00, 0.04	-	
	PFO	1	0.00	0.00, 0.35	-	
Age						0.614
	≥60	5	0.02	0.00, 0.05	0.00%	
	<60	2	0.03	0.00, 0.16	-	
Gender (male)						**0.045**
	≥50	2	0.09	0.01, 0.21	-	
	<50	5	0.00	0.00, 0.04	0.00%	
CHA_2_DS_2_-VASc score						0.884
	≥2	6	0.02	0.00, 0.06	0.00%	
	<2	1	0.00	0.00, 0.35	-	
HAS-BLED score						**0.033**
	≥3	4	0.06	0.01, 0.15	0.00%	
	<3	3	0.00	0.00, 0.04	-	
Follow-up (months)						0.088
	>12	3	0.05	0.01, 0.11	-	
	≤12	4	0.00	0.00, 0.02	0.00%	

Statistically significant *p* values have been blackened. ASD, atrial septal defect; PFO, patent foramen ovale.

#### 3.3.2 Device-Related Events 

**Fig. 5.**
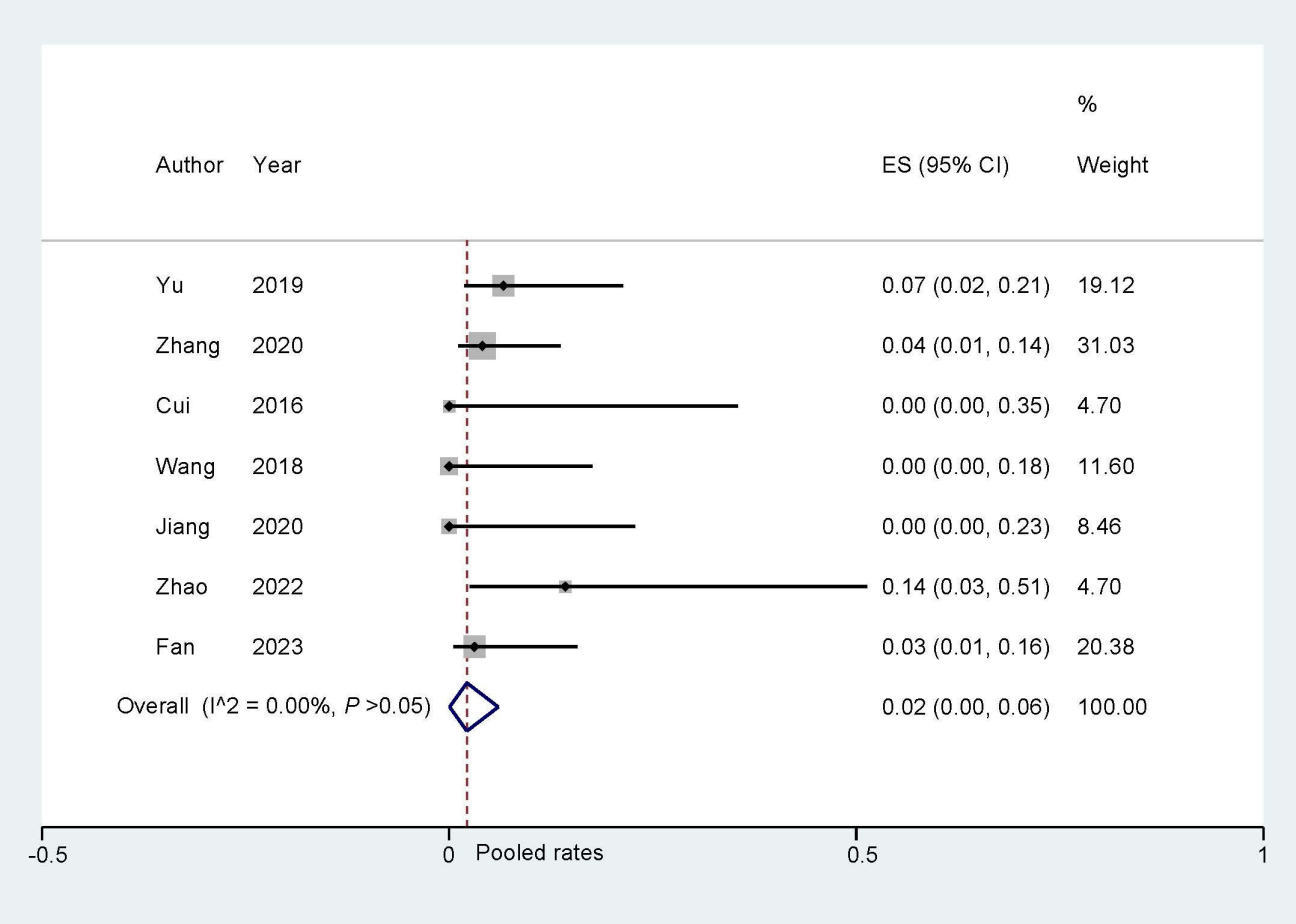
**Device-related events during follow up**. ES, effect size.

The pooled device-related events that occurred during the follow-up were analyzed by a fixed effect model. The pooled rate was found to be 0.02 (95% CI: 0.00, 0.06; I^2^ = 0.00%; Fig. 5). Sensitivity analysis was carried out, and the findings indicated that there were no significant alterations in the overall combined proportion, ranging from 0.05 (95% CI: 0.02, 0.10) to 0.70 (95% CI: 0.03, 0.16). Additionally, Egger’s test demonstrated no evidence of publication bias (*p* = 0.981). Simultaneously, the subgroup analysis was conducted simultaneously, and the results are presented in Table 4. Overall, the incidence of device-related events did not show significant differences among subgroups in the ‘One stop’ ASD/PFO closure with LAAC.

**Table 4.** **The subgroup analysis for device-related events**.

Factors		Study number	Pooled incidence	95% CI	I^2^ (%)	*p* value
Sample size						0.536
	≥20	3	0.04	0.01, 0.09	-	
	<20	4	0.00	0.00, 0.07	0.00%	
Interatrial communications types						0.437
	ASD and PFO	4	0.02	0.00, 0.07	0.00%	
	ASD	2	0.02	0.00, 0.09	-	
	PFO	1	0.14	0.03, 0.51	-	
Age						0.330
	≥60	5	0.03	0.00, 0.08	0.00%	
	<60	2	0.00	0.00, 0.07	-	
Gender (male)						0.535
	≥50	2	0.04	0.00, 0.14	-	
	<50	5	0.02	0.00, 0.06	0.00%	
CHA_2_DS_2_-VASc score						0.818
	≥2	6	0.03	0.00, 0.07	0.00%	
	<2	1	0.00	0.00, 0.35	-	
HAS-BLED score						0.660
	≥3	4	0.02	0.00, 0.10	0.00%	
	<3	3	0.03	0.00, 0.08	-	
Follow-up (months)						0.963
	>12	3	0.03	0.00, 0.09	-	
	≤12	4	0.01	0.00, 0.08	0.00%	

ASD, atrial septal defect; PFO, patent foramen ovale.

#### 3.3.3 All-Cause Death

**Fig. 6.**
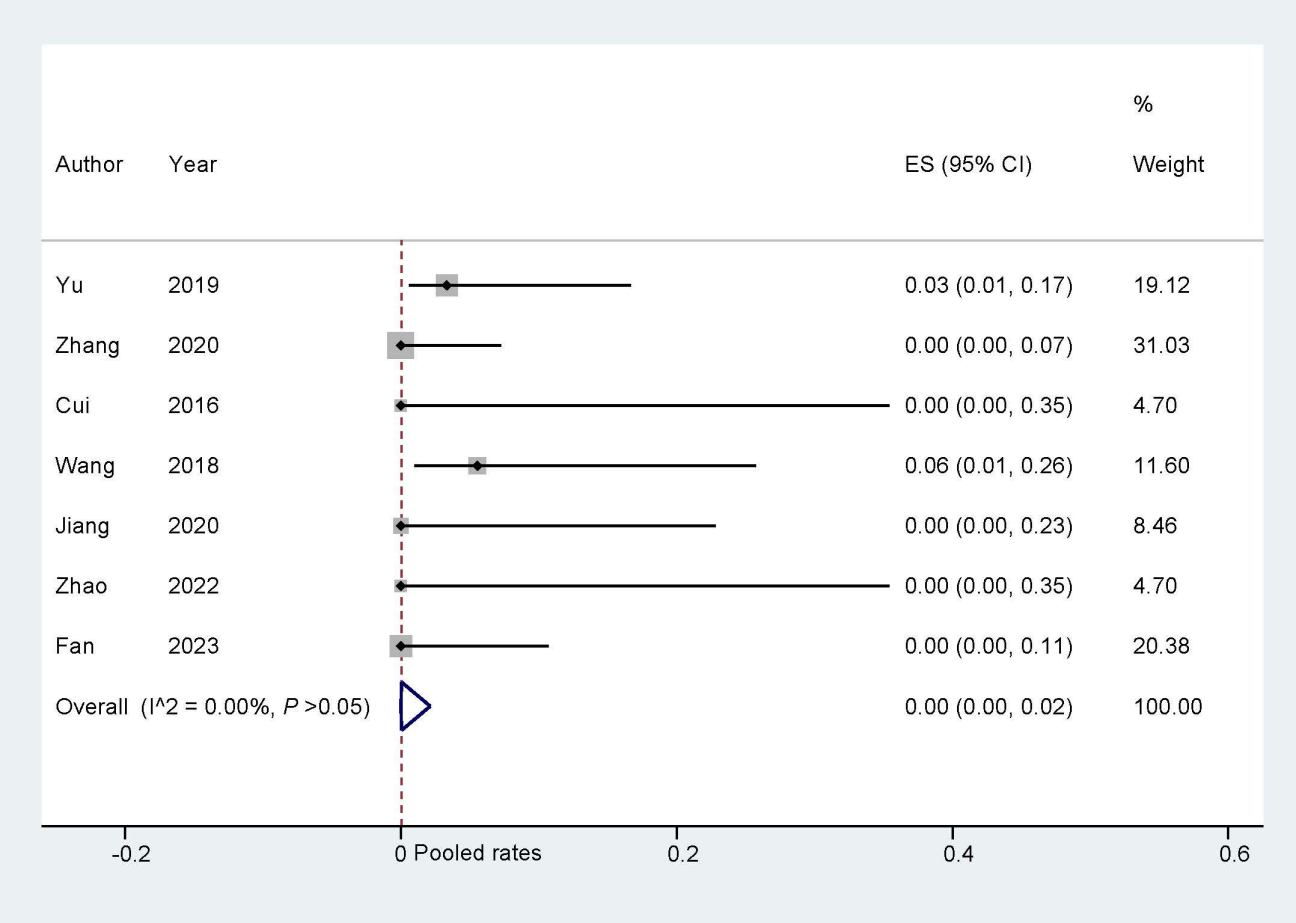
**All-cause death during follow-up**. ES, effect size.

The analysis of all cause deaths from the included studies was conducted using a fixed model. The overall incidence results suggest that the ‘One stop’ ASD/PFO closure with LAAC is associated with very low all-cause mortality (Fig. 6). Meanwhile, sensitivity analysis showed that the pooled proportion and heterogeneity, ranged from –4.81 (95% CI: –15.29, 5.66) to 1.26 (95% CI: –8.55, 11.06), revealing no single study dominated the combined proportion. Moreover, Egger’s test indicated no evidence of publication bias (*p* = 0.371). indicating the robustness of the results. A total of 7 subgroup factors were selected for subgroup analysis, and the results are displayed in Table 5. In the HAS-BLED score ≥3 subgroup, the risk of all-cause death showed an upward trend, while there was no statistical difference noted (*p* = 0.078).

**Table 5.** **The subgroup analysis for all cause death**.

Factors		Study number	Pooled incidence	95% CI	I^2^ (%)	*p* value
Sample size						0.293
	≥20	3	0.00	0.00, 0.03	-	
	<20	4	0.01	0.00, 0.08	0.00%	
Interatrial communications types						0.797
	ASD and PFO	4	0.00	0.00, 0.04	0.00%	
	ASD	2	0.00	0.00, 0.04	-	
	PFO	1	0.00	0.00, 0.35	-	
Age						0.223
	≥60	5	0.00	0.00, 0.02	0.00%	
	<60	2	0.03	0.00, 0.16	-	
Gender (male)						0.333
	≥50	2	0.01	0.00, 0.10	-	
	<50	5	0.00	0.00, 0.02	0.00%	
CHA_2_DS_2_-VASc score						0.831
	≥2	6	0.00	0.00, 0.02	0.00%	
	<2	1	0.00	0.00, 0.35	0.00%	
HAS-BLED score						0.078
	≥3	4	0.02	0.00, 0.09	0.00%	
	<3	3	0.00	0.00, 0.02	-	
Follow-up (months)						0.666
	>12	3	0.01	0.00, 0.05	-	
	≤12	4	0.00	0.00, 0.02	0.00%	

ASD, atrial septal defect; PFO, patent foramen ovale.

## 4. Discussion

We comprehensively evaluated a total of 156 patients from 7 original articles. Compared with previously published meta-analyses, we implemented more stringent screening criteria [21]. Our focus was on studies that presented findings regarding the efficacy and safety of combined ASD/PFO closure with LAAC in AF patients. Moreover, we reviewed multiple publications from the same trial or cohort and identified the study with the highest patient inclusion. The primary findings are summarized as follows: (1) The combination of ASD/PFO closure with LAAC demonstrates a notably high success rate. (2) ‘One stop’ closure exhibits a high level of long-term safety and a low occurrence of associated complications. (3) In the male proportion ≥50% and HAS-BLED score >3 subgroup, the incidence of bleeding events was relatively higher in ASD/PFO closure combined with LAAC.

Cardiogenic stroke has been reported to account for 25% of ischemic stroke worldwide, which often results in significant risks of high disability and mortality, seriously jeopardizing patients’ lives and health [22]. Furthermore, AF and interatrial communication play a leading role in cardiac stroke. Therefore, preventing strokes in patients with AF and ASD/PFO is of primary importance.

Oral anticoagulation is a well-established and widely accepted approach that effectively mitigates the occurrence of thromboembolic events in AF patients. However, this carries an increased susceptibility to bleeding complications. Recently, LAAC has emerged as a viable mechanical intervention for preventing thromboembolic events while minimizing bleeding risk [23]. Osmancik *et al*. [24, 25] followed patients with a high risk for stroke and increased risk of bleeding at both short-term (19 months) and long-term (4 years) levels and found that LAAC remains noninferior to new oral anticoagulants (NOACs) for preventing neurological, major cardiovascular, or bleeding events. Furthermore, LAAC significantly reduced nonprocedural bleeding.

Sun *et al*. [26] investigated the association between the presence of ASD/PFO and atrial vulnerability and found that atrial septal abnormalities were linked to a 2fold increase in the risk of atrial vulnerability among ischemic stroke patients. Previous studies have shown that in patients with ASD/PFO, atrial shunting increases the volume load on the heart, and long-term high load leads to myocardial fibrosis of the right atrium, structural and electrical remodeling, and promotes the occurrence of AF [27]. For AF patients with ASD/PFO, the older age and larger atrial size make the success rate of radiofrequency ablation low and the recurrence rate high. To prevent the occurrence of AF and stroke, closure of atrial septal defects is recommended for patients with congenital heart disease presenting with ASD/PFO.

However, for AF patients with ASD/PFO, closure of ASD/PFO alone does not significantly reduce thromboembolic complications [28]. Persistent AF remains a major contributor to thromboembolic events, requiring long-term oral anticoagulation therapy. Meanwhile, performing a split operation not only prolongs hospitalization but also significantly increases associated costs. On the other hand, the closure of ASD/PFO leaves an occluder umbrella at the atrial septal site, which makes subsequent interventional procedures for AF difficult to perform. Therefore, for AF patients complicated with ASD/PFO, ‘One stop’ ASD/PFO closure with LAAC can avoid the difficulty of atrial septal puncture in the long term after simple ASD/PFO closure, and to avoid the risk of bleeding caused by long-term anticoagulation therapy, and improve the long-term safety of patients after operation. It provides a novel treatment for AF patients with ASD/PFO. It is worth noting that after performing LAAC, a 10-minute observation was performed to ensure the stability of the occluder umbrella to prevent the occurrence of occluder detachment after ASD/PFO closure [20].

Whereas, there are still insufficient studies on the feasibility, safety and effectiveness of ‘One stop’ interventional therapy for ASD/PFO closure combined with LAAC. Our study illustrates that ‘One stop’ closure is highly safe and effective, with a success rate of 1. It may be an optimal option for the prevention of stroke and other thrombotic complications in non-valvular atrial fibrillation (NVAF) patients with ASD/PFO.

In addition, outcomes of ‘One stop’ ASD/PFO closure combined with LAAC were analyzed as well. During the follow-up period, the incidences of cerebrovascular events, thromboembolic events, and all-cause death were notably low at 0.00 and the incidence of bleeding events and device-related events was 0.02, indicating a promising long-term prognosis and reliable safety performance. Bleeding events after ‘One stop’ ASD/PFO closure with LAAC may be closely related to postoperative antithrombotic regimens. However, optimal antithrombotic therapy after ‘One stop’ closure is not well established as no randomized evaluation has been performed to date. The current recommended antithrombotic regimen following LAAC involves 45 days of OACs or warfarin in combination with aspirin, followed by 4.5 months on dual antiplatelet therapy involving aspirin and clopidogrel. Subsequently, aspirin alone should be continued indefinitely from 6 months post-implantation onward [29, 30]. The anticoagulation strategies in the studies we included predominantly followed this regimen (**Supplementary Material 2**). Further research is needed to comprehensively evaluate the optimal postoperative antithrombotic strategy.

Subgroup analyses were also performed for individual long-term adverse events. Notably, in the male proportion ≥50% subgroup and HAS-BLED score ≥3 subgroup, ASD/PFO closure combined with LAAC demonstrated increased bleeding events (*p* = 0.045 and *p* = 0.033, respectively). A previous cohort study indicated that the efficacy of OACs treatment in individuals with AF was comparable between male and female, as there were no significant differences observed in complications such as systemic embolism or stroke. However, it was noted that males had a significantly higher risk of major bleeding compared to females [31]. While Nakashima* et al*. [32] demonstrated that the absence of prior catheter ablation, a history of major bleeding, and baseline anemia were independent predictive factors for late bleeding events after LAAC and sex did not impact post-LAAC bleeding outcomes. Meanwhile, Zhao* et al*. [33] found no association between gender and post-LAAC bleeding. The impact of gender on bleeding events during ‘one-stop’ closure has not been thoroughly investigated to date. Our results preliminarily indicate a higher risk of bleeding with ‘one-stop’ closure in male patients. However, further research is required to validate this finding.

The HAS-BLED score is widely utilized for the assessment of bleeding risk in patients with AF during anticoagulant therapy [34]. The HAS-BLED score is influenced by various factors, including hypertension, abnormal liver and kidney function, stroke, etc. rendering it a dynamic assessment tool for patients. Concurrently, research has demonstrated a positive correlation between high HAS-BLED scores and an elevated risk of bleeding and a HAS-BLED score ≥3 indicates a significant propensity for bleeding. Our study also demonstrated a positive correlation between higher HAS-BLED scores and an increased risk of bleeding following the ‘One stop’ closure. Hence, according to the current latest guidelines for AF management, it is imperative for AF patients to optimize the risk factors in the HAS-BLED score prior to undergoing ‘One stop’ closure [34].

## 5. Limitations

Several limitations should be emphasized. First, studies enrolled in this meta-analysis are nonrandomized, observational design, thus selection bias cannot be completely ruled out. Second, a total of 7 articles were included, and 2 of which had a sample size of less than 10, which might have affected the distribution of the results. Moreover, only 3 studies had a follow up period of more than 12 months, adding to the instability of the long-term complication results of the ‘One stop’ closure. In addition, ‘One stop’ ASD/PFO closure combined with LAAC is more complicated than conventional surgery. The success rate of surgery is also affected by different regions, hospitals and medical teams.

## 6. Conclusions

Our study suggests that ‘One stop’ ASD/PFO closure combined with LAAC is effective and safe for AF patients with interatrial communication.

## Availability of Data and Materials

The data which support the findings of this study are available from the corresponding author upon reasonable request.

## Supplementary Material

Supplementary material associated with this article can be found, in the online version, at https://doi.org/10.31083/RCM26662.
