# Supplementary material for: ‘One Stop’ Therapy has a Satisfying Performance on AF Patients with Interatrial Communication: Evidence from Pooled Clinical Experience
Source: Rev Cardiovasc Med. 2025 Apr 16;26(4):26662. doi: 10.31083/RCM26662 (PMC12059784; doi:10.31083/RCM26662)
Supplement: Supplementary file 1 [file 2153-8174-26-4-26662-s1.zip › Supplementary Material 1.docx]

As the ASD/PFO closure is a relatively cutting-edge technique, it has not yet been incorporated into the relevant Medical Subject Headings (MESH) terms, rendering it unsearchable in MESH. The relevant published literature and commonly used abbreviations were searched as the search strategy to ensure a comprehensive review.

**Pubmed**

#1 ("Atrial Fibrillation"[Mesh]) OR "Atrial Fibrillations" OR "Fibrillation, Atrial" OR "Fibrillations, Atrial" OR "Auricular Fibrillation" OR "Auricular Fibrillations" OR "Fibrillation, Auricular" OR "Fibrillations, Auricular" OR "Persistent Atrial Fibrillation" OR "Atrial Fibrillation, Persistent" OR "Atrial Fibrillations, Persistent" OR "Fibrillation, Persistent Atrial" OR "Fibrillations, Persistent Atrial" OR "Persistent Atrial Fibrillations" OR "Familial Atrial Fibrillation" OR "Atrial Fibrillation, Familial" OR "Atrial Fibrillations, Familial" OR "Familial Atrial Fibrillations" OR " Fibrillation, Familial Atrial" OR "Fibrillations, Familial Atrial" OR "Paroxysmal Atrial Fibrillation" OR "Atrial Fibrillation, Paroxysmal" OR "Atrial Fibrillations, Paroxysma" OR "Fibrillation, Paroxysmal Atrial" OR "Fibrillations, Paroxysmal Atrial" OR "Paroxysmal Atrial Fibrillations" OR "AF" OR "atrial fibrillation" OR "non-valvular atrial fibrillation" OR "NVAF" **192899**

#2 ("left atrial appendage closure"[Mesh]) OR " LAA Closure" OR " Closure, LAA" OR " LAA Closures" OR " Left Atrial Appendage Occlusion Intervention" OR "LAAC" OR "LAA Closure" OR "left atrial appendage occlusion" OR "LAAO" **3012**

#3 "patent foramen ovale closure" OR "PFO closure" OR " patent foramen ovale occlusion" OR "PFO occlusion" OR "atrial septal defect closure" OR "ASD closure" OR "atrial septal defect occlusion" OR "ASD occlusion" OR "PFO/ASD closure" **3344**

#4 #1 AND #2 AND #3 **23**

**Cochrane Library**

#1 MeSH descriptor: [Atrial Fibrillation] explode all tree **7431**

#2 (Atrial Fibrillations):ti,ab,kw OR (Fibrillation, Atrial):ti,ab,kw OR (Fibrillations, Atrial):ti,ab,kw OR (Auricular Fibrillation):ti,ab,kw OR (Auricular Fibrillations):ti,ab,kw OR (Fibrillation, Auricular):ti,ab,kw OR (Fibrillations, Auricular):ti,ab,kw OR (Persistent Atrial Fibrillation):ti,ab,kw OR (Atrial Fibrillation, Persistent):ti,ab,kw OR (Atrial Fibrillations, Persistent):ti,ab,kw OR (Fibrillation, Persistent Atrial):ti,ab,kw OR (Fibrillations, Persistent Atrial):ti,ab,kw OR (Persistent Atrial Fibrillations):ti,ab,kw OR (Familial Atrial Fibrillation):ti,ab,kw OR (Atrial Fibrillation, Familial):ti,ab,kw OR (Atrial Fibrillations, Familial):ti,ab,kw OR (Familial Atrial Fibrillations):ti,ab,kw OR ( Fibrillation, Familial Atrial):ti,ab,kw OR (Fibrillations, Familial Atrial):ti,ab,kw OR (Paroxysmal Atrial Fibrillation):ti,ab,kw OR (Atrial Fibrillation, Paroxysmal):ti,ab,kw OR (Atrial Fibrillations, Paroxysma):ti,ab,kw OR (Fibrillation, Paroxysmal Atrial):ti,ab,kw OR (Fibrillations, Paroxysmal Atrial):ti,ab,kw OR (Paroxysmal Atrial Fibrillations):ti,ab,kw OR (AF):ti,ab,kw OR (atrial fibrillation):ti,ab,kw OR (non-valvular atrial fibrillation):ti,ab,kw OR (NVAF):ti,ab,kw **19277**

#3 #1 OR #2 **19277**

#4 MeSH descriptor: [Left Atrial Appendage Closure] explode all trees **1**

#5 (LAA Closure):ti,ab,kw OR ( Closure, LAA):ti,ab,kw OR ( LAA Closures):ti,ab,kw OR ( Left Atrial Appendage Occlusion Intervention):ti,ab,kw OR (LAAC):ti,ab,kw OR (LAA Closure):ti,ab,kw OR (left atrial appendage occlusion):ti,ab,kw OR (LAAO):ti,ab,kw **350**

#6 #4 OR #5 **351**

#7 (patent foramen ovale closure):ti,ab,kw OR (PFO closure):ti,ab,kw OR (patent foramen ovale occlusion):ti,ab,kw OR (PFO occlusion):ti,ab,kw OR (atrial septal defect closure):ti,ab,kw OR (ASD closure):ti,ab,kw OR (atrial septal defect occlusion):ti,ab,kw OR (ASD occlusion):ti,ab,kw **427**

#8 #3 AND #6 AND #7 **1**

**Web of Science**

#1 TS=(("Atrial Fibrillation"[Mesh]) OR "Atrial Fibrillations" OR "Fibrillation, Atrial" OR "Fibrillations, Atrial" OR "Auricular Fibrillation" OR "Auricular Fibrillations" OR "Fibrillation, Auricular" OR "Fibrillations, Auricular" OR "Persistent Atrial Fibrillation" OR "Atrial Fibrillation, Persistent" OR "Atrial Fibrillations, Persistent" OR "Fibrillation, Persistent Atrial" OR "Fibrillations, Persistent Atrial" OR "Persistent Atrial Fibrillations" OR "Familial Atrial Fibrillation" OR "Atrial Fibrillation, Familial" OR "Atrial Fibrillations, Familial" OR "Familial Atrial Fibrillations" OR " Fibrillation, Familial Atrial" OR "Fibrillations, Familial Atrial" OR "Paroxysmal Atrial Fibrillation" OR "Atrial Fibrillation, Paroxysmal" OR "Atrial Fibrillations, Paroxysma" OR "Fibrillation, Paroxysmal Atrial" OR "Fibrillations, Paroxysmal Atrial" OR "Paroxysmal Atrial Fibrillations" OR "AF" OR "atrial fibrillation" OR "non-valvular atrial fibrillation" OR "NVAF") and Preprint Citation Index (Exclude – Database) **245486**

#2 TS=(("left atrial appendage closure"[Mesh]) OR " LAA Closure" OR " Closure, LAA" OR " LAA Closures" OR " Left Atrial Appendage Occlusion Intervention" OR "LAAC" OR "LAA Closure" OR "left atrial appendage occlusion" OR "LAAO") and Preprint Citation Index (Exclude – Database) **4133**

#3 TS=("patent foramen ovale closure" OR "PFO closure" OR " patent foramen ovale occlusion" OR "PFO occlusion" OR "atrial septal defect closure" OR "ASD closure" OR "atrial septal defect occlusion" OR "ASD occlusion" OR "PFO/ASD closure") **4424**

#4 #1 AND #2 AND #3 and Preprint Citation Index (Exclude – Database) **31**

**Embase**

#1 'atrial fibrillations' OR 'auricular fibrillation'/exp OR 'auricular fibrillation' OR 'auricular fibrillations' OR 'persistent atrial fibrillation'/exp OR 'persistent atrial fibrillation' OR 'persistent atrial fibrillations' OR 'familial atrial fibrillation'/exp OR 'familial atrial fibrillation' OR 'familial atrial fibrillations' OR 'paroxysmal atrial fibrillation'/exp OR 'paroxysmal atrial fibrillation' OR 'paroxysmal atrial fibrillations' OR 'af' OR 'atrial fibrillation'/exp OR 'atrial fibrillation' OR 'non-valvular atrial fibrillation'/exp OR 'non-valvular atrial fibrillation' OR 'nvaf' **314848**

#2 'left atrial appendage closure'/exp OR 'left atrial appendage closure' OR 'closure, laa' OR 'laa closures' OR 'left atrial appendage occlusion intervention' OR 'laac' OR 'laa closure'/exp OR 'laa closure' OR 'left atrial appendage occlusion'/exp OR 'left atrial appendage occlusion' OR 'laao' **7880**

#3 'patent foramen ovale closure' OR 'pfo closure' OR 'patent foramen ovale occlusion' OR 'pfo occlusion' OR 'atrial septal defect closure' OR 'asd closure' OR 'atrial septal defect occlusion' OR 'asd occlusion' OR 'pfo/asd closure' **6093**

#4 #1 AND #2 AND #3 **69**

**CNKI**

SU='心房颤动'+'心房纤颤'+'房颤'+'非瓣膜性房颤' AND SU='左心耳封堵术'+'左心耳封堵'+'经皮左心耳封堵术' AND SU='卵圆孔未闭'+'卵圆孔未闭封堵术'+'卵圆孔未闭封堵'+'房间隔缺损'+'房间隔缺损封堵术'+'房间隔缺损封堵' **15**

**WANFANG**

("心房颤动" OR "心房纤颤" OR "房颤" OR "非瓣膜性房颤") AND ("左心耳封堵术" OR "左心耳封堵" OR "经皮左心耳封堵术") AND ("卵圆孔未闭" OR "卵圆孔未闭封堵术" OR "卵圆孔未闭封堵" OR "房间隔缺损" OR "房间隔缺损封堵术" OR "房间隔缺损封堵") **23**

**The excluded reports before determining the finial eligible studies:**

**4 reviews**

Calabrò P, Gragnano F, Niccoli G, et al. Antithrombotic Therapy in patients undergoing transcatheter interventions for structural heart disease. Circulation. 2021;144(16):1323-1343.

Freixa X, Arzamendi D, Tzikas A, et al. Cardiac procedures to prevent stroke: patent foramen ovale closure/left atrial appendage occlusion. Can J Cardiol. 2014;30(1):87-95.

Yap J, Chen S, Stripe BR, et al. Transseptal access for left heart structural interventions in the setting of prior atrial septal defect closure. Catheter Cardiovasc Interv. 2020;95(3):414-419.

Mitsis A, Kyriakou M, Christodoulou E, et al. Antithrombotic therapy following structural heart disease interventions: current status and future directions. Rev Cardiovasc Med. 2024;25(2):60.

**1 meta-analysis**

1.Song Y, Xing H, Koch PD, et al. The feasibility and safety of combining atrial septal defect/patent foramen ovale and left atrial appendage closure: a systematic review and meta-analysis. Front Cardiovasc Med. 2023;9:1080257.

**11 case-reports**

1.Yap J, Chen S, Stripe BR, et al. Transseptal access for left heart structural interventions in the setting of prior atrial septal defect closure. Catheter Cardiovasc Interv. 2020;95(3):414-419.

2.Meier B, Tarbine SG, Costantini CR. Percutaneous management of left atrial appendage perforation during device closure. Catheter Cardiovasc Interv. 2014;83(2):305-7.

3.Song S, Lee OH, Kim JS,et al. Simultaneous closure of a left atrial appendage through an atrial septal defect and the atrial septal defect. Yonsei Med J. 2017;58(6):1237-1240.

4.Çöteli C, Canpolat U, Kaya EB,et al. Left atrial appendage closure using Amulet device in a patient with prior percutaneous atrial septal defect closure. Turk Kardiyol Dern Ars. 2018;46(4):306-308.

5.Matta M, Maltese L, Ugo F, et al. Feasibility and safety of left atrial appendage closure in a patient with previous foramen ovale occlusion: a case report. Eur Heart J Case Rep. 2021;5(3):ytab113.

6.Ahmad S, Goldsweig AM. Left atrial appendage occlusion and patent foramen ovale closure using a steerable sheath and intracardiac echocardiography. Catheter Cardiovasc Interv. 2024;104(2):416-418.

7.Wu YC, Wang MX, Chen GC,et al. Cryoballoon pulmonary vein isolation and left atrial appendage occlusion prior to atrial septal defect closure: a case report. World J Clin Cases. 2022;10(12):3872-3878.

8.Chen YG, Wu P, Zhao X, Wu LY, Bai P, Zhang H, Ren P. Permanent atrial fibrillation is restored and sinus rhythm maintained without catheter ablation after atrial septal defect closure: a case report. Heart Surg Forum. 2022;25(5):E676-E679.

9.Korsholm K, Jensen JM, Nielsen-Kudsk JE. Left atrial appendage occlusion guided by intracardiac echocardiography in a patient with a 34 mm atrial septal defect occluder: a case report. Eur Heart J Case Rep. 2023;7(12):ytad571.

10.Pan CL, Zhong YC, Mao XB, et al. Percutaneous coronary intervention + left atrial appendage occlusion+patent foramen ovale occlusion "one-stop treatment of atrial fibrillation with coronary heart disease and high risk of stroke: a case report. Chin J Intervent Cardiol.2022;30(01):61-63.

11.Zhu ML, Zhang H, Zhang XL,et al. Atrial septal defect with atrial fibrillation treated by "one-stop" operation of left atrial auricle occlusion plus room absence occlusion: a case report. J Prac Electrocardiol JS.2020;02:143-144.

**1 unavailable to extract**

1.Kleinecke C, Fuerholz M, Buffle E, et al. Transseptal puncture versus patent foramen ovale or atrial septal defect access for left atrial appendage closure. EuroIntervention. 2020;16(2):e173-e180.

**3 duplicate studies**

1.Jiang XF, Zhong LT, Wang K, et al. Safety and efficacy of one-stop treatment of left atrial appendage occlusion in combination with concomitant closure of atrial septum defect or patent foramen ovale.South China J of Cardiology.2021.1:36-41.

2.Jiang XF, Zhu WM, Li Jun,et al.Preliminary study on the safety and efficacy of one-stop treatment of percutaneous left atrial appendage closure combined with coronary intervention for patients with atrial fibrillation complicated with coronary heart disease.Chin J of Cardiovascular Review.2019.01:73-75

3.Wang JM, Zhu XY, Wang QG,et al.Effect analysis of transcatheter left atrial ear closure in patients with atrial septal traffic and atrial fibrillation.Chin Circul J.2017.32:156

**2 sample size <5**

1.Leong MC, Kandavello G, Husin A, et al. Left atrial appendage and atrial septal occlusion in elderly patients with atrial septal defect and atrial fibrillation. Pacing Clin Electrophysiol. 2020 ;43(11):1252-1257.

2.Gafoor S, Franke J, Boehm P,et al Leaving no hole unclosed: left atrial appendage occlusion in patients having closure of patent foramen ovale or atrial septal defect. J Interv Cardiol. 2014;27(4):414-22.

**1 the subjects not patients with AF**

1.Kuwata S, Vierecke J, Gloekler S, et al. Left atrial appendage closure for "primary primary" prevention during percutaneous closure of septal defects in patients with large atria but no atrial fibrillation. Cardiol J. 2018;25(2):179-187.
