# Supplementary material for: ‘One Stop’ Therapy has a Satisfying Performance on AF Patients with Interatrial Communication: Evidence from Pooled Clinical Experience
Source: Rev Cardiovasc Med. 2025 Apr 16;26(4):26662. doi: 10.31083/RCM26662 (PMC12059784; doi:10.31083/RCM26662)
Supplement: Supplementary file 1 [file 2153-8174-26-4-26662-s1.zip › Supplementary Material 2.docx]

**Table1 Summary of the indications for one-stop procedure, devices used during the procedure and postoperative antithrombotic therapy.**

| **First author (year)** | **Indications** | **LAAC related devices** | **PFO related devices** | **Postoperative regimen** |
| --- | --- | --- | --- | --- |
| Yu (2019) | NVAF patients with contraindication of long term OAC coexisting with congenital interatrial septal communications (PFO/ASD) | Watchman device | the Amplatzer PFO occlude or IrisFIT occlude or Figulla FlexII occlude or the Amplatzer ASD occluder | Post-implantation LAAC drug regimen was either Warfarin if no contraindication, or combined enoxaparin with aspirin if contraindication to Warfarin till 45 days. Then the patient was switched to both aspirin and clopidogrel until 6 months and eventually aspirin alone. |
| Zhang (2020) | (1). For patients with PFO, they have ischemic stroke, or transient ischemic attack (TIA), or a peripheral thromboembolic event or a large right-to-left shunt.For patients with ASD, TTE show clear indications for ASD occlusion. (2). TEE show clear indications for LAAC. (3).Non-valvular AF. (4). CHA2DS2VASc score ≥2, and HAS-BLED score ≥3 or having a contraindication to anticoagulant therapy or unwilling to receive long-term warfarin therapy.  (5). Patients were willing to accept the one-stop occlusion. | Watchman device | Cardi-O-Fix Occluder | Oral warfarin or Novel Oral Anticoagulants (NOAC) was administered at 45-60 days after operation. If the TEE showed complete closure of the LAA, no device-related thrombus, the patients were then switched to both aspirin and clopidogrel until 6 months. After 6 months, they began taking aspirin tablets (100 mg) once per day. |
| Cui (2016) | Nonvalvular permanent atrial fibrillation patients with contraindication of long-term warfarin coexisting with congenital interatrial septal communications (PFO/ASD) | The Amplatzer™ Cardiac Plug | The type of device was not specified | Patients were given aspirin 100mg and clopidogrel 75mg for 60 days after operation, and aspirin 100mg daily for the rest of their lives. |
| Wang (2018) | (1). Nonvalvular AF patients aged ≥18 year and the CHA_2_DS_2_VASc score ≥1.  (2). Patients with contraindications to warfarin application and HAS-BLED bleeding risk score ≥3.  (3). Patients with congenital ASD/PFO.  (4). Aspirin and clopidogrel can be taken orally after operation. | The LACBES® occluder or the Amplatzer™ Cardiac Plug | SHSMA ASD/PFO occluder | Patients were given aspirin 100mg and clopidogrel 75mg for 60 days after operation, and aspirin 100mg daily for the rest of their lives |
| Jiang (2020) | (1). Secundum atrial septal defect.  (2). Nonvalvular atrial fibrillatio and the duration of atrial fibrillation more than 1 year.  (3). Age >18 years old.  (4). CHA_2_DS_2_-VASc score ≥2. | Watchman device | The type of device was not specified | Patients were given rivaroxaban till 45 days  Then the patient was switched to both aspirin and clopidogrel until 6 months and eventually aspirin alone. |
| Zhao (2022) | (1). Age ≥18 years old.  (2). Meeting the diagnostic criteria for atrial fibrillation.  (3). Meeting the diagnostic criteria of PFO.  (4). The patient met the indication of left atrial appendage occlusion. | Watchman device or the LACBES® occluder or LAmbre device | Cardi-O-Fix Occluder or MemoSorb | Anticoagulation and antiplatelet therapy during perioperative period and after discharge were performed according to Chinese expert consensus on left atrial appendage closure to prevent stroke in patients with atrial fibrillation (2019). |
| Fan (2023) | (1). Non-valvular persistent atrial fibrillation, onset time of atrial fibrillation > 3 months;  (2). Age >18 years old.  (3). CHA2DS2-VASc score ≥2.  (4). HAS-BLED score ≥3 points, or still have embolism under long-term anticoagulation therapy, or radiofrequency ablation is considered to have poor therapeutic effect by team evaluation, and easy to relapse after operation.  (5). Contraindications to warfarin use. | The type of device was not specified | HeartR™ device | Patients were given rivaroxaban till 3 months.  Then the patient was switched to both aspirin and clopidogrel until 6 months and eventually aspirin alone. |
